# Evaluation of the Memory Effect on Gold-Coated Silica Adsorption Tubes Used for the Analysis of Gaseous Mercury by Cold Vapor Atomic Absorption Spectrometry

**DOI:** 10.1155/2013/763893

**Published:** 2013-03-25

**Authors:** Mohammad Mahmudur Rahman, Richard J. C. Brown, Ki-Hyun Kim, Hye-On Yoon, Nhu-Thuc Phan

**Affiliations:** ^1^Department of Environment and Energy, Sejong University, 98 Gun-Ja Dong, Gwang-Jin Gu, Seoul 143-747, Republic of Korea; ^2^Analytical Science Division, National Physical Laboratory, Hampton Road, Teddington TW11 0LW, UK; ^3^Korea Basic Science Institute, 126-16, 5th St., Anam-dong, Sungbuk-ku, Seoul 136-701, Republic of Korea

## Abstract

In an effort to reduce the experimental bias involved in the analysis of gaseous elemental mercury (Hg^o^), the blank response from gold-coated adsorption tubes has been investigated using cold vapor atomic absorption spectrometry (CVAAS). Our study has been compared with our recent investigation on memory effect in a cold vapour atomic fluorescence spectrometry (CVAFS). The pattern of blank responses was quantified after loading different amounts of mercury and after different time intervals of 1, 14, and 45 days. In case of the one day interval, the result of five to six instant blank heating cycles confirmed successful liberation of mercury following the second and third blank heating cycles. The results of 14 or 45 days generally suggest that liberation of excess mercury is affected by both the initial loading amount and the length of storage time prior to analysis. We have demonstrated a possibly effective way to reduce memory effects. Some similarities of these results with those from CVAFS experiment suggests that the blank response is caused by a combination of mercury absorbed within the bulk gold and micro- and nanoparticles liberated during heating and not from coabsorbing interfering gaseous species.

## 1. Introduction

 At present, mercury in our environment is originating mainly from anthropogenic sources such as burning of fossil fuels and gold mining activities, accounting for about 45% and 18%, respectively [1]. To better understand the behaviour of mercury within the atmospheric cycle, speciation is often crucial. In ambient air, mercury species are dominated by gaseous rather than particulate-bound components. Gaseous mercury is usually classified into three categories: (1) elemental mercury, (2) inorganic mercury, and (3) organic mercury [2]. 

Although there is a conceptual difference between the two terms, gaseous elemental mercury (GEM: Hg^o^) and total gaseous mercury (TGM), they have often been used interchangeably because of the dominance of GEM over other species [3]. GEM is known to be the predominant component of gaseous Hg (>95% and often >99%) with a large atmospheric life span (1 month to 1.5 years) [4, 5]. The lifespans of the remaining airborne mercury species such as gaseous oxidized mercury (GOM) also called gaseous reactive mercury, particle bound mercury (Hg_p_), and organic mercury tend to be short (e.g., between one to seven days). As such, they can be subject to rapid settlement in lower atmosphere via wet and dry deposition very near their sources [4–8]. Many previous investigations relying on modelling tools and field data have suggested that GOM generated by the oxidation of GEM in the free troposphere is an important mechanism of Hg input to terrestrial ecosystems [9–12]. 

Until now, various measurement methodologies have been developed to determine accurate GEM concentrations in ambient air. To collect GEM, gold amalgamation (trapping and desorption) is the most common choice [13]. In general, during air sampling for subsequent GEM analysis, ambient air is passed through adsorption tubes filled with high surface area gold particles (gold-coated quartz sand), where mercury is trapped by an amalgamation mechanism. The adsorption tubes are then subsequently analysed by spectrometric methods, especially cold vapor atomic absorption spectrometry (CVAAS) and cold vapor atomic fluorescence spectrometry (CVAFS), which can achieve high sensitivities, down to a few tens of picogram (pg) or less [13]. The benefits of the adsorption tube method include its ability to lower of the overall detection limit because of preconcentration, while also enabling the collection of remote samples for centralized analysis [14]. Comparisons between CVAAS and CVAFS for mercury analysis generally show good comparability with low experimental biases, especially if interfering species are absent [13].

In a previous investigation the selection of sampling volume was found to affect experimental bias because of its association with recovery [15]. Furthermore, short- and long-term memory effects in the analysis of adsorption tubes are one of the critical sources of experimental biases in quantification [14]. Whilst these memory effects have been quantified for CVAFS, no such study exists for CVAAS. Such a comparable study would reveal more information about the nature of the memory effect, because of the different mechanisms of operation of the two analysis techniques. Therefore, we designed a series of experiments to allow evaluation of the memory effect in the analysis of gaseous Hg. Through a modification of experimental design used in our previous study [14], we attempted to learn more about the short- and long-term memory effect for CVAAS analysis. In this study, we additionally aimed to characterize memory effect through the extension of storage intervals and with different initial loading amounts of Hg. By comparing the properties of the memory effect between the present and previous study, we aim to learn more about the fundamental characteristics of the effect. 

## 2. Experimental Assembles

### 2.1. Basic Setups

**Figure 1:**
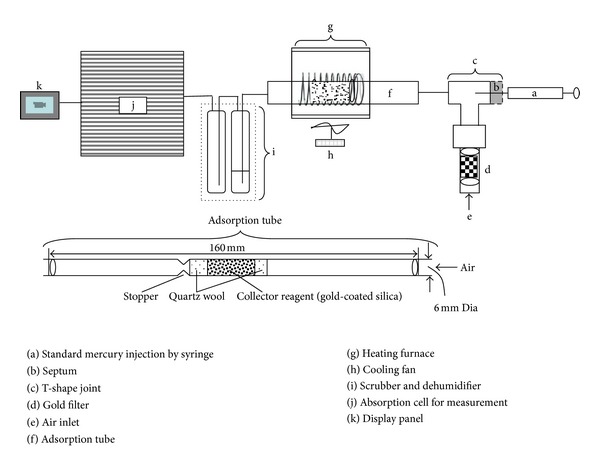
Schematic diagram of instrumental settings and composition of adsorption tube used for the analysis of elemental Hg in our study.

 In our study, we conducted four different types of experiment to understand the sensitivities of the memory effect to variations in storage time and initial mass of Hg loaded onto the tube. To this end, a total of 12 gold-coated sand tubes were used, and the ID for each individual is given in Table 1. Gold-coated sand (part number: 03115), used for making adsorption tubes (Figure 1), was brought from Brooks Rand Labs. Following the procedures described in the operational manual of the mercury analyzer with CVAAS detector (WA-4, NIC, Japan), adsorption tubes were prepared as follows. Glass tubes (160 mm in length and 6 mm in diameter (inner)) were used with a crimp in the middle of the tube to hold the quartz wool and gold coated sand in place. Then, hollow tubes were filled with gold-coated sand and quartz wool (Figure 1). After making these adsorption tubes, they were tested by injecting a known amount of gaseous mercury standard. Following these tests, the trap was then desorbed to its blank level [16]. 

The mercury detector was calibrated against known concentrations of mercury gaseous standard before each experiment. In our experiment, we injected between 5 and 50 ng of mercury from a Standard Gas Box (MB-1, NIC, Japan) into the injection port of analyzer (Figure 1). Sample adsorption tubes were placed in the outside port of the Hg analyzer and heated to 600°C for 5 minutes, desorbing the mercury from trap and into the CVAAS detector (Figure 1). Good linearity was observed (calibration coefficient of determination (*r* ^2^ = 0.99)) for each calibration and consecutive experiment (Table 1). 

### 2.2. Experimental Design

In this study, four different types of experiments were conducted to precisely evaluate blank memory behaviour of amalgamation tube method on the basis of CVAAS detection. As this study aims to describe reproducibility of the sampling method, all the basic conditions of two different studies are compared. As a first step, the experimental setups used in this study and our previous study [14] have been summarized in Table 2. The major differences between these two studies are the analytical system and standard gas box. In the previous study of Brown et al. [14], atomic fluorescence spectrometry (10.525 Galahad analyzer) and bell-jar for standard were used, respectively. The calibration apparatus (bell-jar versus standard gas box) should make no difference to the results since the calibration mass will still be traceable to the Dumarey equation for the saturated mass concentration of mercury in air [17]. The experimental schemes used in both studies are summarized in Table 3. Exp. 1 was aimed at understanding the short-term memory effect, while Exp. 2 and 3 aimed to elucidate the intermediate-term memory effect. In Exp. 4, experiments were conducted to test the memory effect over the longest duration of up to 45 days. In case of Exp. 1, four different masses (5, 10, 30, and 50 ng) of standard mercury were taken from the gas box and injected into the adsorption tubes. All of these standard samples were analyzed instantly using six consecutive heating cycles (1 standard analysis and five consecutive blank runs in a single heating cycle). The prime objective of Exp. 1 was to observe the pattern of instant mercury liberation in the short term with increasing initial loading amount. 

To observe the memory effect over an intermediate-timescale, two types of similar experiments were conducted. In Exp. 2, analysis was made by extending the total storage of samples up to 15 days. In Exp. 2, after running an instant analysis comprising six heating cycles (like Exp. 1), a further six consecutive blank heating cycles were made for each tube, at different intervals of 8 days and 15 days. The objective of Exp. 2, was to observe liberation of any excess mass of mercury owing to the memory effect over intermediate timescales. Although Brown et al. [14] used the same range of standards (5 to 50 ng) in the first investigation (like our Exp. 1), their second investigation (Exp. 2) was confined to only one mass (5 ng). Hence, our data allows assessment of the effect of different mass loadings over an extended period (Table 3). 

In Exp. 3, a different type of intermediate-term memory experiment was carried out. Here, unlike Exp. 2, the tubes were analyzed and left without instant blank treatment. In addition, only 1 blank heating cycle was used instead of 6 (Exp. 2). Hence, a proportion of Hg may remain after the initial analysis. However, similarly to Exp. 2 the 2nd and 3rd heating cycle was made at 8 and 15 days after the first tube analysis. After completing Exp. 3, a total of 8 blank data points were obtained using 12 pre-calibrated tubes (Table 3). Similarly to our previous investigation [14], comparison was made for five different masses (5, 12, 28, 40, and 45) after 8 and 15 days. 

In Exp. 4, after initial loading of three different amounts of mercury (5, 10, and 30 ng), liberation of excess mass was recorded from second and third heating cycle at four different intervals: 1, 7, 14, and 45 days. As two consecutive blank runs were made for the three masses at four interval days, a total of 24 blank data are obtained from Exp. 4 (Table 3). Due to some instrumental errors at higher injection masses (50 ng), we have only recorded the results from three masses. Exp. 4 was conducted to understand the pattern of excess mercury liberation from two consecutive heating cycles with an increasing time gap between initial loading day and blank run day. In case of Brown et al., only one injection mass was analyzed (5 ng) in Table 3. 

 In this study, all measurement data were presented as an average of triplicate analysis. For these experiments, a total number of up to 12 tubes were used in each experiment, as described in Table 3. After injecting standard mercury masses from the standard gas box into analyzer, the sample peak was integrated by the software within the analyzer. To calculate concentration, peaks were divided by the calibration slope for that particular tube. In Tables 2 and 3, the experimental scheme used in this study is compared to our previous study [14].

## 3. Result and Discussion 

### 3.1. Short-Term Memory Effect

**Figure 2:**
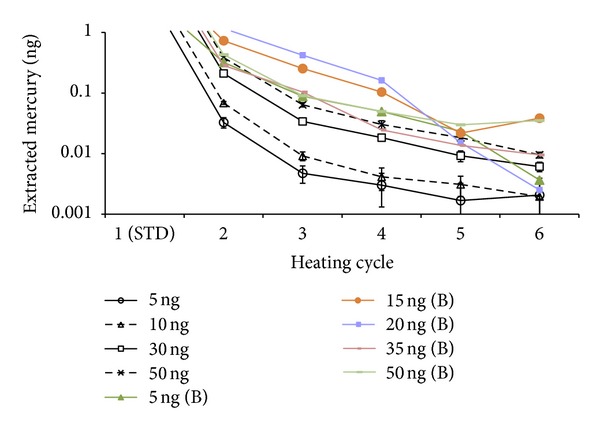
Results of short-term Exp. (Exp. 1). Analytical intensity obtained for each heating cycle during the initial analysis of adsorption tubes dosed with different masses of mercury (5, 10, 30, and 50 ng). In each heating cycle, standard mercury was analyzed first and blank runs were made successively. Each point is average of triplicate analysis. In the legend section, the letter “B” is representing investigation of Brown et al. [14]. 5 to 50 ng (B) represents observation from 2nd heating cycle in our previous investigation [14].

Mercury from gold-coated sand adsorption tubes generally cannot be fully desorbed by a single thermal treatment. The amount of mercury that can be recovered from the first heating cycle is limited by a number of factors that include the initial mass loaded and the duration of thermal treatment. The mercury remaining after the initial heating step can hence be evaluated to account for the memory effect. In our first experiment (Exp. 1), we investigated the short-term memory effect by running 6 consecutive blank heating cycles after dosing the tube from 5 to 50 ng of Hg within the same days. In the CVAAS system, although most of the mercury was liberated in the first heating cycle, excess mercury was liberated subsequently in the following heating cycles. The magnitude of such releases depended on the initial amount of mercury loaded (Figure 2). For tubes loaded with relatively low amounts of mercury (up to 10 ng), which is in the range of most of the ambient air samples, we observed that liberation of the adsorbed mercury was almost complete (99.99%) after the third heating cycle adsorption tubes. 

**Figure 3:**
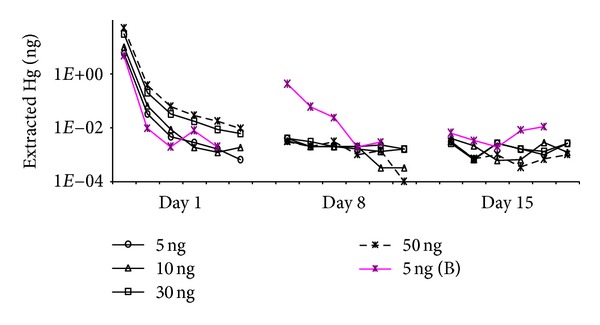
Results of intermediate-term Exp. A (Exp. no. 2). Analytical intensities from adsorption tubes dosed with four different amounts of mercury (5, 10, 30, and 50 ng): six consecutive runs made after (a) 1 day, (b) 8 days, and (c) 15 days. 5 ng (B) represents observation from 2nd heating cycle in our previous investigation [14].

**Figure 4:**
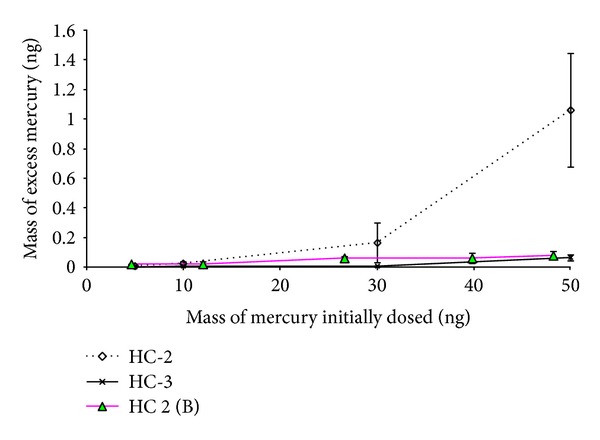
Results of intermediate-term Exp. B (Exp. no. 3). Amount of excess mercury (ng) from the second heating cycle (HC-2) after 7 days and third heating cycle (HC-3) after 14 days was measured: results compared as a function of the mass of mercury originally dosed onto the adsorption tubes in day 1 (*x*-axis). Each measurement point (HC-2 and HC-3) for initial injection of 5, 10, 30, and 50 ng of mercury is average of triplicate tube analyses, and a total of 12 different tubes were used for this investigation. Error bars represent standard deviation of triplicate analyses. HC-2 (B) represents observation from the second heating cycle in our previous investigation [14].

**Figure 5:**
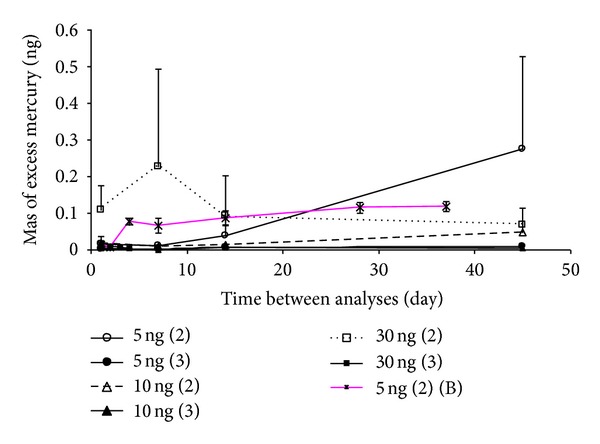
Results of long-term Exp (Exp. no. 4). Amount (ng) of excess mercury measured from the two consecutive blank (the second and third) analyses of adsorption tubes originally loaded with three different amount of mercury (5, 10, and 30 ng). For every standard concentration injection, time gap between first and other (second and third) heating cycles was 1 to 45 days. Each measurement point is the average value of triplicate tube analyses, and a total of nine tubes were used for this analysis. Error bars represent standard deviation of each point (only showing the positive direction). 5 ng (2) (B) represents observation from the second heating cycle in our previous investigation [14].

In figures (Figures 2, 3, 4, and 5), we compared the results of this study with those of our previous investigation [14] (assigned as legend symbol B). In Figure 2, the excess mass of mercury liberated from the 2nd to 6th heating cycle in our previous investigation [14] is fairly high relatively to this study. In this study, the mercury obtained from second heating cycle ranged between 0.64 and 0.74% when the initial loading amount was 5 and 50 ng, respectively. By contrast, during the second heating cycle of a 5 ng loading, Brown et al. [14] found a blank value of 0.309 ng which is about an order of magnitude larger than our values (0.032 ng). However, Brown et al.'s blank data (0.42 ng) at 50 ng standard were similar to the data in this study (0.37 ng). In this study, it was observed that the amount of mercury liberated from subsequent blank heating cycle shows a close correlation with the amount of mercury initially loaded. In Brown et al. [14], the trend of mercury liberation was rather irregular (in Exp. 1), if compared with initial loading mass; at initial loadings of 5 and 50 ng, the second heating cycle liberated 0.31 and 0.42 ng of mercury, respectively. More importantly, in both studies, liberation of excess mass during the 6th blank run decreased dramatically to below 0.009 ng. 

### 3.2. Intermediate-Term Memory Effect

For the study of the memory effect over intermediate timescales, two separate experiments, 2 and 3, were conducted (intermediate types A (Figure 3) and B (Figure 4), respectively (Table 3)) as described above. We did not see any significant extraction (<0.02% of initial loading) of mercury after 8 days and 15 days with and average RSE of blank values in each heating cycle in the range 6%–33% (Table 4). However, at day 1, RSE values from individual heating cycles were above 40% because Hg masses liberated after the first heating cycle were highly irregular (Table 4). More importantly, Pandey et al. [13] found, after the first heating cycle for the tube initially loaded with 5 ng at day 1 (Exp. 2), higher analytical intensity from 2nd (9%) and 3rd (1.02%) blank heating cycle at day 8. However, their results at day 15 were similar to our investigation (0.02% of initial loading mass) (Table 3). Unlike the pattern observed by Brown et al. [14], our investigation suggests that tubes are in good condition for storage with no long-term memory effect for up to 15 days, if adsorption tubes are cleaned rigorously (at least 6 heating cycles) during the first analysis step. As such, the results of our study indicate that the mass liberated as a result of the memory effect over intermediate timescales is fairly insignificant (<0.02%) compared to its initial loading.

In Exp. 3, we investigated memory effect patterns under limited conditioning (e.g., one desorption cycle at each interval up to 15 days). The result of Exp. 3 indicates the possibility that significant Hg blanks can occur as the mass of mercury dosed onto the tube increases, if tube is stored without sufficient conditioning (Figure 4). For the initial loading of 30 ng, the increment in second heating cycle was very low (0.25% of initial loading). However, it tended to peak in the second heating cycle most noticeably to show a 2.12% rise for the maximum initial loading of 50 ng. By contrast, such initial mass dependency was not apparent in Brown et al. [14] although their blank run was made during the second heating cycle. Brown et al. [14] found that the proportion of Hg left on tube was between 0.22 and 1%, when initial injection ranged between 1 to 45 ng (Figure 4). The possible mechanism of this effect was suspected to reflect diffusion of small proportion of mercury into the bulk gold of adsorption tube. Although it may not occur during sampling, such accumulation of Hg may occur during the desorption stage at elevated temperature [14]. 

### 3.3. Long-Term Memory Effect

In Exp. 4, the pattern of Hg liberation was investigated based on the blank runs during the 2nd and 3rd heating cycle up to a prolonged period of 45 days (Table 3). In this investigation, the effect of the initial loading mass was observed clearly in the second cycle, as the largest blank in the second cycle appeared with 30 ng initial loading. Although we wanted to include a point at 50 ng for this comparative calibration, we did not do so due to significant system contamination at these high masses. We have thus limited the mass range of Hg into 3 different masses in Exp. 4. 

It is interesting to find that with extension of storage period mercury liberation can continue to occur up to the 2nd heating cycle (Figure 5). When the blank run interval was elongated, the blank level of even the lowest loadings of 5 ng peaked significantly in the 2nd heating cycle. Its liberation surged dramatically from 0.017 (±0.003)  ng at day 1 to 0.28 (±0.01) ng at day 45. This increasing trend at 5 ng is different from other masses tested in this study as well as those of Brown et al. [14] (Table 3), who did not observe any significant blank effect from almost all storage durations tested, except the first blank run (on day 1). Because the previous investigation of Brown et al. [14] only focused on a single dose of 5 ng, some additional information may be inferred from this long-term storage effect. The result of the 45-day expriment in this study suggests that the extended storage may provide an extra source of bias, regardless of the initial mass loading. However, such effect was unlikely to occur when tubes were stored for less than 20 days of storage or after the second heating cycle. In a previous study, although field samples for TGM were collected routinely using gold-coated quartz sand and analyzed using CVAFS [18], long-term memory effects were not considered. 

In Figures 4 and 5, it can be said with adequate confidence that the 2nd heating cycle is one of the key processes required to liberate excess mercury under laboratory conditions. In the previous investigation of Brown et al. [14], the possible mechanisms behind the long-term memory effect were described elaborately, and these can be considered in this study as well. However, according to Sabri et al. [19], mercury may be retained for a longer period of time on rough gold surfaces with large number of monolayer coverages owing to its strong affinity. In addition, Sabri et al. [20] found that higher energy is needed to separate mercury from a rough gold surface compared to polished one. These combined effects can be responsible for producing memory effects as adsorption tubes contain a gold coating on quartz sand which is rough at the microscale. 

### 3.4. Strategy to Reduce Experimental Bias Associated with Memory Effect

The results of our study consistently indicate that one should consider performing at least 2nd, 3rd, and 4th blank heating cycles after analyzing more than 5 ng of mercury to avoid memory effects on subsequent use of the tubes. Hence, for instance, if we calculate excess masses detected from the heating cycle of Exp. 1, we can use this data to achieve enhanced analytical accuracy. In case of 50 ng of initial injection, liberation of excess mass in the 1st, 2nd, 3rd, and 4th blank heating cycles amounted to 0.46 ng ( = 0.37 + 0.062 + 0.029 + 0.018). The sum of these blank runs is about 1% of the initial loading amount of Hg. As such, considering our measured data, we can write down the following equation by following the procedure of Brown et al. [14]:

(1) $$\begin{array}{c} M_{t}=M_{\text{STD}}+M_{1}+M_{2}+M_{3}+M_{4}-5M_{5}, \end{array}$$

where *M*_*t*_ is Final mass, *M*_STD_ is Mass of standard injection, *M*_(2−5)_ representes Mass from the 2nd to 5th heating cycle, and 5 *M*_5_ is Five times of *M*_5_ (this value has been subtracted as a finite but stable tube blank).

 More importantly, the above equation (1) may not be fixed for all measurements, as tubes may sometimes liberate excess mass of mercury even after five blank heating runs, depending on their previous history or the storage duration of the adsorption tube. As such, according to our study, operators should observe the pattern of analytical responses and gauge whether the finite tube blank has yet been reached; that is, in our system machine blank mass values should be ≤0.01 ng. Either way results can be resolved systematically, if we use proposed correction (1) or its more general form given below, which may be employed under all circumstances:

(2) $$\begin{array}{c} M_{t}=M_{\text{STD}}+M_{1}+M_{2}+M_{3}+M_{4}+\cdots +M_{n-1}-nM_{n},\;\; \end{array}$$

where *M*_*n*−1_ and *M*_*n*_ represent the response from the (*n* − 1)th and *n*th heating cycles, respectively. The value of *n* should be chosen such that the operator is sure the finite tube blank has been reached.

 In Exp. 2 as part of the intermediate-term blank memory effect investigation, we did not observe any significant increment of blank levels after day 8 and day 15. Thus, it can be said that stored blank tubes should have been maintained under good condition providing that enough blank cycles were run following initial analysis. By contrast, according to Brown et al. [14], tubes should be cleaned at day 8 to minimize memory effect, as they found elevated level of mercury at that time (about 0.42 ng in the first heating cycle). 

The results of Exp. 3 suggest that up to 30 ng of initial loading, liberation of excess mass from the second heating cycle (at day 8) was below 0.53%. By contrast, the increment at day 8 was about seven times higher for the 50 ng initial loading. Brown et al. [14] measured an effect in the range between 0.16 and 0.21% at day 7 for initial loadings in excess of 25 ng. However, in both studies, liberation of excess mass during the third heating cycle was consistently insignificant (<0.02%), except at 50 ng (Figure 4). Overall, the combination of low standard injection masses and the liberation of any remaining excess mass from second heating cycle can enhance analytical accuracy significantly. Considering this effect, it is not good practice to keep tube for prolonged period of time without a second blank run step. It should be noted that, in this investigation, when we analyzed mass at 50 ng or above 50 ng, the system was contaminated and internal cleanup was needed to stabilize the system. To measure accurately at these high masses tubes should be cleaned rigorously after analysis using repeated blank runs to maintain blank level mass below 0.01 ng in CVAAS system. 

If we keep tubes for long periods of time (about 45 days) after standard or real sample analysis without intermittent tube cleaning, excess Hg mass will be liberated. This excess mass will increase as the time gap between analysis increases (Figure 5). Such effects can be significant, as the pattern became highly unpredictable after long storage periods. 

## 4. Conclusion

In this study, to quantify memory effect patterns of mercury adsorption tubes containing gold-coated quartz sand on short (1 day), intermediate (15 days), and long (45 days) timescales, liberation of excess mass of gaseous mercury from blank heating cycles has been investigated by CVAAS system. Due to fairly limited previous work on the memory effect, this study has been designed to allow direct comparison with our previous investigation [14] using CVAFS. In this research, four different types of experiments were conducted to quantify the memory effect over different time cycles. Although most of the mercury (≥99%) was liberated in the first heating cycle after injecting standard mercury masses between 5 to 50 ng, the addition of five subsequent blank heating cycles liberated the remaining mercury (referred to as the short-term memory effect).

Although the mechanism behind memory effect in adsorption tubes was not well understood, experimental bias can be reduced by understanding the pattern of excess mass of Hg liberated from blank heating cycles. In addition, to reduce experimental bias, adsorption tubes should be cleaned by considering the number of blank heating cycles required to reach the finite tube blank level. Most importantly, if one needs to minimize the experimental bias due to the memory effect, it is necessary to conduct at least five blank heating cycles. Because of the relatively small amount of data available on this topic, in particular in this work and our previous study [14], further investigation is necessary to better understand the mechanism controlling the memory effect in Hg analysis. However, the similarities in the memory effect observed in these two studies suggest that it is originating from the same source—deep absorption of mercury within the gold substrate. The specific differences observed in this study as compared to our previous one are most probably a function of the difference sorbent materials used in the tubes. This is another indication that such sorbent materials will need to be characterised on a case by case basis. The difference in operation between the CVAFS and CVAAS techniques also reveals some extra information. Because no increase in the extent of the short- and long-term memory effects was observed when using the CVAAS this suggests that interfering compounds (such as hydrocarbons and sulphur species), which are visible to CVAAS but not to CVAFS, are unlikely to be involved in the memory effect. Furthermore, since both analytical techniques are sensitive to particles and the different adsorption tubes used displayed differences in long-term memory effects, the observation of finite tube blank levels over and above the response observed for injection of blank gas in the absence of an absorption tube in both studies suggests that the liberation of micro- and nanosized particles during heating cycles is responsible for this effect rather than the continued liberation of small quantities of mercury.

## Figures and Tables

**Table 1:** Basic information concerning the adsorption tubes used in our study.

Order	Tube ID	Injected mass (ng) during Exp.^a^	Calibration results	
			*R* ^2^	Slope^b^
1	XF		0.997	1.02
2	F	5	0.998	1.06
3	K		0.998	1.02
4	C		0.998	0.99
5	D	10	0.994	1.01
6	E		0.996	1.05
7	J		0.998	1.06
8	KM	30	0.996	1.03
9	L		0.998	0.93
10	H		0.998	1.06
11	D	50^c^	0.999	1.08
12	I		0.999	1.06

^a^Masses were injected in each of all four experiments.

^b^Slope values were calculated using zero offset.

^c^50 ng data were not obtained in Exp. 4 (due to system contamination).

**Table 2:** The experimental settings used in this study and a previous study (Brown et al., 2011 [14]).

Order	Parameters	This study	Brown et al. [14]
1	Analyzer	Mercury Analyzer, WA-4 (NIC, Japan)	10.525 Sir Galahad analyzer
2	Place of analysis	Atmospheric Environmental Laboratory, Sejong University.	National Physical Laboratory (NPL), UK.
3	Detector	Cold vapor atomic absorption spectrometry (CVAAS)	Atomic fluorescence spectrometry (AFS)
4	Gold-coated sand	Brooks Rand Labs (part number: 03115)	Amasil, PS Analytical, UK
5	Calibration software	Integrated with WA-4 analyzer	NPL's XLGENLINE
6	Used mercury in Exp.	5 to 50 ng	5 to 50 ng
7	Standard gas box	Standard gas box (MB-1), NIC, Japan.	Bell-jar calibration vessel (PS Analytical, part number: G523V002)

**Table 3:** Comparison of the detailed experimental design for short- and long-term memory effects of Hg sampler and differences between the two studies considered.

Order	Exp. ID	Exp. name	Major Exp. parameters	This study	Brown et al. [14]	Relative comparison
			(a) Amount of STD Hg (ng) injected	5, 10, 30, and 50	5, 15, 20, 35, and 50	In both studies, findings were similar, maximum mercury was liberated in the first heating cycle, and tubes were almost blank by the 6th heating cycle. However, liberation of excess mass from 2nd BHCs was about 0.39 (±0.20) ng higher in the investigation of Brown et al.
			(b) Blank heating cycles (BHC)	5^a^ (day 0)	6	
			(c) Daily interval between STD and blank analysis	None (1-day study)	None (1-day study)	
1	Exp. 1	Short-term memory effect	(d) Total tube used	4 mass × 3 tube (triplicate analysis per mass) = 12	5 mass × 5 tube (five times analysis per mass) = 25	
			(e) Number of blank run^b^	5^a^ (consecutive run (3 tube/mass)) × 4 (mass) = 20	5 (consecutive run (5 tube/mass)) × 5 (mass) = 25	
			(f) Findings	Majority of Hg was liberated in the first three heating cycles and was almost blank by the 6th heating cycle. For higher masses, more heating cycles were needed.	Tubes were almost blank by the 5th heating cycle, irrespective of initial mass loading.	

			(a) Amount of STD Hg (ng) injected	5, 10, 30, and 50	5	Four different masses of mercury were injected in our study, while Brown et al.'s study used only one mass and found liberation of excess mass at day 8, which was absent in our study.
			(b) BHC	6 (days 1, 8, and 15)	4 (days 1, 8, and 15)	
			(c) Total duration (no. of days)	15	15	
2	Exp. 2	Intermediate-term A	(d) Total tube used	4 mass × 3 tube (triplicate analysis per mass) = 12	1 mass × 5 tube (five mass analysis per mass) = 12	
			(e) Number of blank run^b^	6 (consecutive blanks (3 tube/mass)) × 4 (mass) × 3 (interval days) = 72	5 (consecutive blanks (3 tube/mass)) × 1 (mass) × 3 (interval days) = 15	
			(f) Findings	Day 1 analysis was similar to Exp. 1, while insignificant blank level at day 8 and 15.	There were some memory effects at day 8, especially in the first three heating cycle. However, day-15 tubes were blank.	

			(a) Amount of STD Hg (ng) injected	5, 10, 30, and 50	5, 12, 28, 40, and 45	In our study, after 20 ng initial injection, gradual increment was seen. In contrast, Brown et al. found linear increment from the beginning.
			(b) BHC	1 (days 8 and 15)	1 (days 8 and 15)	
			(c) Total duration (no. of days)	15	15	
			(d) Total tube used	4 mass × 3 tube (triplicate analysis per mass) = 12	1 mass × 5 tube (five times analysis per mass) = 5	
3	Exp. 3	Intermediate-term B	(e) Number of blank run data^b^	1 (blank (3 tube/mass)) × 4 (mass) × 2 (interval days) = 8	1 (blank (5 tube/mass)) × 5 (mass) × 2 (interval days) = 10	
			(f) Findings	Up until 10 ng of initial injection, average of 2nd analysis was around blank level. Then the liberation increased gradually until 30 ng. After that, sharp increment was seen. Consistently 3rd heating cycle was around blank level throughout the study.	Linear increment was seen with increasing initial mass of mercury, irrespective of initial STD injection.	

			(a) Amount of STD Hg (ng) injected	5, 10, and 30	5	Similarly to Exp. 2, we used more initial standard injections. Brown et al. showed linear and steady state condition; however, we found different pattern for different initial injections. In both studies, similar trends were seen from third heating cycle.
			(b) BHC	2 (days 1, 8, 15, and 45)	2 (days 1, 2, 4, 7, 14, 28, and 35)	
			(c) Total duration (no. of days)	45	35	
			(d) Total tube used	3 mass × 3 tube (triplicate analysis per mass) = 9	1 mass × 5 tube (five times analysis per mass) = 5	
4	Exp. 4	Long term	(e) Number of blank run data^b^	2 (blank (3 tube/mass)) × 3 (mass) × 4 (interval days) = 24	2 (blank (3 tube/mass)) × 1 (mass) × 7 (interval days) = 14	
			(f) Findings	Initial 5 ng injection showed high memory effect in 2nd heating cycle which was increased with reanalysis time, while higher injection mass (30 and 50 ng) showed lower liberation amount with time. During 3rd analysis tubes were almost blank over the study period.	In 2nd heating cycle, up to 20 days interval increment was linear with time. After that increment was steady. Third heating cycle was near to the blank level.	

^a^Out of 6 consecutive runs, only 5 blank runs (except the 1st standard analysis) are counted in Exp 1.

^b^To count the number of blank run data only, all the standards measured in the 1st run are not counted in intermediate- and long-term experiments (Exps. 2, 3, and 4).

**Table 4:** Relative standard error (RSE) of blank heating cycles in intermediate-term type A study (Exp. no. 2).

Order	Interval days	Blank heating cycles						Mean
		1	2	3	4	5	6	
Mean mass (ng)^b^								

1	1	—^a^	0.168	0.027	0.013	0.007	0.004	0.044
2	8	0.004	0.002	0.002	0.002	0.001	0.001	0.002
3	15	0.003	0.002	0.002	0.001	0.001	0.002	0.002

RSE (%)								

1	1	—^a^	45.92	48.75	50.41	52.46	44.11	48.33
2	8	6.91	11.21	11.71	13.87	18.50	14.25	12.74
3	15	10.04	19.22	23.30	20.29	33.21	22.88	21.49

^a^Initial standard injection mass values were not included.

^b^Liberation of mean excess masses for blank runs (triplicate) from four different injection amounts of 5, 10, 30, and 50 ng.
